# Co‐Occurrence of Apathy and Impulsivity in Progressive Supranuclear Palsy

**DOI:** 10.1002/mdc3.13339

**Published:** 2021-09-16

**Authors:** Zi Q. Kok, Alexander G. Murley, Timothy Rittman, James Rowe, Luca Passamonti

**Affiliations:** ^1^ School of Clinical Medicine Cambridge University Hospitals Cambridge United Kingdom; ^2^ Department of Clinical Neurosciences University of Cambridge Cambridge United Kingdom; ^3^ Cognition and Brain Sciences Unit University of Cambridge Cambridge United Kingdom; ^4^ Istituto di Bioimmagini e Fisiologia Molecolare, Consiglio Nazionale Ricerche Milan Italy

**Keywords:** apathy, impulsivity, progressive supranuclear palsy

## Abstract

**Background:**

Apathy and impulsivity are common consequences of progressive supranuclear palsy (PSP) and can worsen its prognosis. They can co‐exist in the same patients although their concomitant prevalence remains unclear. Their relationship to emotional lability is unknown.

**Objectives:**

To estimate the co‐occurrence of apathy and impulsivity and their relationship to emotional lability in PSP. To characterize the demographic, clinical, and cognitive features of PSP patients with apathy and impulsivity.

**Methods:**

In a retrospective study of a long‐term clinical cohort, we assessed the prevalence of apathy, impulsivity, and emotional lability from clinical interviews, medical records, and contemporary carer questionnaires. One hundred fifty‐four patients with a diagnosis of probable or possible PSP (according to the 2017 Movement Disorder Society criteria) were identified. Sixty‐four of these patients had neuropathological confirmation of PSP. PSP patients with both apathy and impulsivity were compared in terms of demographic, clinical, and cognitive characteristics to PSP patients with either one or neither of these neuropsychiatric features.

**Results:**

Apathy and impulsivity co‐existed in two‐thirds of people with PSP. A fifth displayed emotional lability in addition to apathy and impulsivity. Apathy and impulsivity were more commonly co‐expressed than by chance. There was no single demographic, clinical or cognitive feature that distinguished between PSP patients with versus patients without apathy and impulsivity.

**Conclusions:**

The co‐existence of apathy and impulsivity in PSP suggests that these neuropsychiatric features may share similar risk factors and etio‐pathogenetic mechanisms. Apathy and impulsivity should be jointly assessed when planning symptomatic treatments for detrimental behavioral problems caused by PSP.

Progressive supranuclear palsy (PSP) is a neurodegenerative disorder caused by neuronal and glial aggregation of hyperphosphorylated 4‐R tau isoforms, as part of the spectrum of diseases caused by frontotemporal lobar degeneration.^1^ PSP is characterized by postural instability, akinetic rigidity, and oculomotor dysfunction.^1^ Patients with PSP often display prominent neuropsychiatric problems, including apathy and impulsivity.^2^, ^3^, ^4^ In PSP and related disorders, apathy and impulsivity reduce survival^5^ and predict loss of functional independence.^6^

Apathy and impulsivity are common, multifactorial, and frequently co‐existent symptoms. Apathy has affective, cognitive, and behavioral components,^7^, ^8^ with a resultant loss of interest in activities and difficulty initiating actions.^9^ Impulsivity encompasses actions that are premature, without foresight of deleterious consequences or made as a result of a failure to inhibit contextually inappropriate responses.^10^, ^11^ Apathy and impulsivity tend to occur together in PSP,^4^, ^12^ frontotemporal dementia,^11^ and Parkinson's disease.^13^

This co‐existence of apathy and impulsivity argues against the motivational spectrum hypothesis, with apathy and impulsivity at opposite ends of the spectrum.^14^, ^15^ There are several and not necessarily mutually exclusive explanations for the reported co‐existence of apathy and impulsivity. First, it may be chance, given that both are common. Second, they may have a shared neuroanatomical basis,^12^, ^16^ including the degeneration of analogous fronto‐striato‐thalamo‐cortical circuits, which represent actions, rewards, and inhibitory control.^11^ Third, there may be shared neurochemical systems mediating apathy and impulsivity,^11^, ^17^ which would be especially relevant for pharmacological treatments. In clinical practice, dopaminergic agents do not clearly ameliorate apathy or impulsivity,^11^ but serotonergic and noradrenergic strategies might be effective.^18^, ^19^, ^20^ Fourth, individuals may express apathy and impulsivity at different times, perhaps alternating between them. Detailed temporal studies are lacking, but if the few actions made by an apathetic individual were premature, high‐risk and contextually disinhibited, this would be best interpreted as truly concurrent apathy and impulsivity.

Despite many studies of apathy and impulsivity in PSP,^2^, ^4^, ^12^, ^16^, ^21^, ^22^, ^23^, ^24^ basic questions about their co‐existence in PSP remain unanswered. For example, is their co‐existence more frequent than by chance? To what extent do these behavioral problems relate to other contextually inappropriate behaviors, such as emotional lability (or “incontinence”)? Emotional lability refers to sudden, rapid, exaggerated, and uncontrollable bursts of laughing or crying that are spontaneous and often inappropriate to the social context. During these episodes, patients may report congruent emotional sentiments (ie, feeling sad while crying) or an incongruity between the external emotional appearance and their “internal” feelings (ie, not feeling sad while crying).^25^ Emotional lability has been associated to lesions in the pontine nuclei, cerebellum, and frontal lobes,^26^ but a link to impulsivity, apathy or both has not been investigated.

This study has three aims. First, to test the hypothesis that apathy and impulsivity co‐exist in PSP, at the level of individual patients. Second, to test the association between presence of emotional lability and apathy or impulsivity. Third, to compare demographic, clinical, and cognitive features across PSP patients with or without apathy and impulsivity.

## Methods

### Participants

This study included a sample of 154 patients with a clinical diagnosis of probable or possible PSP, identified by retrospective re‐diagnosis according to the 2017 Movement Disorder Society (MDS) criteria.^1^ The data were extracted from research records and electronic medical records at a tertiary referral centre, from the Pick's Disease and Progressive Supranuclear Palsy Prevalence and Incidence protocol (12/EE/0475), the “Diagnosis and Prognosis in PSP and CBD” protocol (07/q0102/3) and the Prospective Evaluation of Parkinson's Plus and Related Disorders Protocol (07/Q0102/3). Sixty‐four patients had pathological confirmation of PSP, via the Cambridge Brain Bank. Two patients were excluded from the study as they received a pathological diagnosis that differed from their clinical diagnosis of PSP. Exclusion criteria were the following: diagnosis of other neurodegenerative conditions including corticobasal syndrome (although the PSP‐CBS subtype as per the 2017 MDS criteria was included), primary akinesia of gait freezing (PAGF), Lewy body dementia, idiopathic Parkinson's disease, Alzheimer's disease, stroke, cancer, and normal pressure hydrocephalus.

Clinical and demographic features included gender, age at symptom onset, disease duration, and PSP subtype.^1^ Disease severity, motor symptoms, cognition, and behavioral problems were respectively assessed via the Progressive Supranuclear Palsy‐Rating Scale (PSPRS), Unified Parkinson's Disease Rating Scale (UPDRS‐part III), Addenbrooke's Cognitive Examination‐Revised (ACE‐R), Frontal Assessment Battery (FAB), and Cambridge Behavioral Inventory‐revised (CBI‐R). Behavioral changes were also identified throughout qualitative analysis of clinical letters and notes, coded in binary terms (absent or present) based on descriptions of apathy, impulsivity, and emotional lability, as reported in Appendix S1. In order for these behavioral changes to be encoded, patients were required to have three or more qualitative features of apathy, impulsivity or emotional lability, and these features had to be reported consistently across three or more clinical follow‐ups. The assessment of behavioral changes was supplemented by scores in the CBI‐R (questions on *Motivation* and *Abnormal behavior)* and PSPRS (questions on withdrawal and irritability, see Appendix S3 for details). Clinical letters were available in all patients. PSPRS was available in n = 79 patients, CBI in n = 52 patients. Twenty‐seven patients completed both CBI and PSPRS. For these patients, apathy and impulsivity were coded as present or absent if the binary coding was fully concordant across the two scales. For patients with either CBI or PSPRS, the binary coding of behavioral abnormalities from the clinical letters had a 100% concordance rate to the items endorsing apathy and impulsivity in the CBI and PSPRS. This is illustrated in Appendix S5. The distribution of CBI scores endorsing apathy and impulsivity is illustrated in Appendix S4.

The Progressive Supranuclear Palsy‐Rating Scale (PSP‐RS) is a 28‐item clinical scale measuring the overall disease severity. It assesses clinical impairment across functional, motor and behavioral domains, and scores patients from 0 to 100. The UPDRS is a clinical scale used to evaluate the severity of both motor and non‐motor aspects of Parkinson's disease. In this study, UPDRS motor scores are used to quantify the severity of PSP patients' motor symptoms. The motor section of the UPDRS scale scores patients from 0 to 52, across 13 subsections. The Addenbrooke's Cognitive Examination‐Revised (ACE‐R) assesses the degree of cognitive impairment by quantifying patient performance in the following domains—attention/orientation, memory, verbal fluency, language, and visuo‐spatial functions. This brings the maximum possible ACE‐R score to 100. The Frontal Assessment Battery (FAB) quantifies the degree of frontal lobe dysfunction by assessing various aspects of executive functions. FAB scores range from 0 to 18, with higher scores indicating better executive functions. On the other hand, the Cambridge Behavioral Inventory (CBI) is a 45‐item informant‐based questionnaire designed to assess neuropsychiatric symptoms in neurodegenerative disorders. It quantifies the severity of behavioral symptoms based on their frequency. CBI scores range from 0 to 180 (with higher scores reflecting a higher level of neuropsychiatric impairment).

We used the ACE‐R and FAB scores recorded within 3 months of the first clinical record of apathetic or impulsive behavioral changes. PSP patients without this information recorded within 3 months of their first behavioral presentation were excluded from the analysis of ACE‐R and FAB scores (n = 35). For patients who experienced neither behavioral changes, we employed the scores measured within 3 months of their diagnosis of PSP. People who were unable to complete the assessments, or those lacking ACE‐R/PSPRS/FAB measures were also excluded from the analysis of the specific clinical scores they lacked (n = 16 patients). Despite being excluded from the analyses that needed the ACE‐R, PSPR or FAB measures, these n = 51 patients were still included in the overall study, including for example the comparison of behavioral changes, demographic variables and other clinical measures.

### Statistical Analyses

Statistical analyses used IBM SPSS (version 27.0) for frequentist analyses and JASP (Version 0.14) for Bayesian analyses. Chi‐squared tests for the co‐occurrence of apathy, impulsivity, and emotional lability were conducted. One‐way analyses of variance (ANOVAs) were used to compare age at onset, disease duration, ACE‐R, FAB, and PSPRS scores (at the first presentation) between groups with both apathy and impulsivity, only apathy or impulsivity or neither of them. Bayesian ANOVAs were used to test the relative evidence of null (no difference) and alternate (difference) hypotheses.

## Results

Patients comprised six subgroups, as shown in Table 1. Most participants (74%) had PSP‐Richardson's syndrome. The remainders spanned PSP with CBS features (10%), PSP with frontal presentation (8%), PSP with primary gait freezing (4%), PSP‐parkinsonism (2%), or PSP with speech and language presentation (1%).

**TABLE 1:** Demographic and clinical data of PSP patients with and without apathy and impulsivity

Variables	Apathy and impulsivity (N = 101)	Impulsivity Alone (N = 15)	Apathy Alone (N = 20)	Neither apathy nor impulsivity (N = 18)	Overall means	F/x̂2	*P*‐values
Demographics							
Gender (% female)	43.6	46.7	45	50	45 (0.5)	‐	0.964
Age at onset	68.1 (7.3)	67.0 (7.4)	68.0 (7.3)	70.3 (6.9)	68.4 (7.22)	0.65	0.585
Pathological confirmation of PSP (%, n)	62.5 (40)	9.4 (6)	14.1 (9)	14.1 (9)	41.6 (64)	‐	0.001
Clinical subtypes, severity and motor features							
PSP‐Richardson's syndrome (%, n)	65.22 (75)	10.43 (12)	14.78 (17)	9.57 (11)	74.68 (115)	‐	<0.001
PSP with CBS features (%, n)	60 (9)	6.67 (1)	20 (3)	13.33 (2)	9.74 (15)	‐	0.001
PSP with predominant frontal presentation (%, n)	92.31 (12)	7.69 (1)	0 (0)	0 (0)	8.44 (13)	‐	0.001
Primary gait freezing variant of PSP (%, n)	42.86 (3)	14.29 (1)	0 (0)	42.86 (3)	4.55 (7)	‐	0.047
PSP‐Parkinsonism (%, n)	33.33 (1)	0 (0)	0 (0)	66.67 (2)	1.95 (3)	‐	0.000
PSP‐SL variant (%, n)	100 (1)	0 (0)	0 (0)	0 (0)	0.65 (1)	‐	0.000
Disease duration (years)	5.29 (2.5)	6.77(4.2)	4.29 (1.8)	5.58 (4.1)	5.34 (2.9)	2.14	0.098
Disease severity (PSPRS scores)	34.92 (13.5)	40.88 (16.7)	28.33 (11.8)	29.63 (13.6)	33.99 (13.8)	1.73	0.168
History (PSPRS scores)	7.16 (3.8)	10.00 (4.8)	6.10 (3.8)	6.00 (4.5)	7.20 (4.0)	1.81	0.153
Mentation (PSPRS scores)	2.78 (2.4)	5.88 (3.9)	1.10 (1.0)	3.00 (2.8)	2.91 (2.7)	5.67	0.002
Bulbar (PSPRS scores)	2.27 (1.4)	2.63 (1.7)	1.60 (1.1)	1.75 (1.0)	2.16 (1.4)	1.20	0.317
Ocular (PSPRS scores)	7.28 (3.8)	7.50 (3.6)	7.78 (3.3)	6.00 (3.1)	7.23 (3.6)	0.39	0.759
Limb (PSPRS scores)	3.68 (2.6)	3.75 (1.2)	3.56 (2.4)	2.75 (2.0)	3.57 (2.4)	0.35	0.788
Gait (PSPRS scores)	10.62 (4.7)	11.13 (5.8)	9.33 (5.7)	10.25 (5.4)	10.48 (4.9)	0.22	0.881
Motor symptoms (UPDRS part III motor scores)	33.09 (12.7)	33.11 (7.7)	28.79 (13.5)	28.86 (12.5)	32.01 (12.3)	0.63	0.601
Cognitive features							
ACE‐R on first assessment of behavioral changes	79.25 (9.3)	75.18 (14.3)	81.80 (11.4)	77.58 (12.2)	74.97 (10.6)	0.91	0.439
FAB on first assessment of behavioral changes	11.66 (3.3)	11.80 (3.0)	13.25 (2.3)	12.00 (1.7)	11.96 (3.1)	0.57	0.639

Means (SDs) are provided. For each of the disease subtypes and pathological confirmation of PSP, percentages of each behavioral subgroup are shown, followed by the number of patients in the corresponding subgroup in brackets that is percentage (number of patients). The p values were corrected for multiple comparisons.

Abbreviations: ACE‐R, Addenbrooke's Cognitive Examination scale‐Revised; CBS, corticobasal syndrome; FAB, Frontal Assessment Battery; PSP‐SL, PSP with speech/language disorders; PSPRS, PSP rating scale; UPDRS, Unified Parkinson's Disease Rating Scale.

**FIG. 1:**
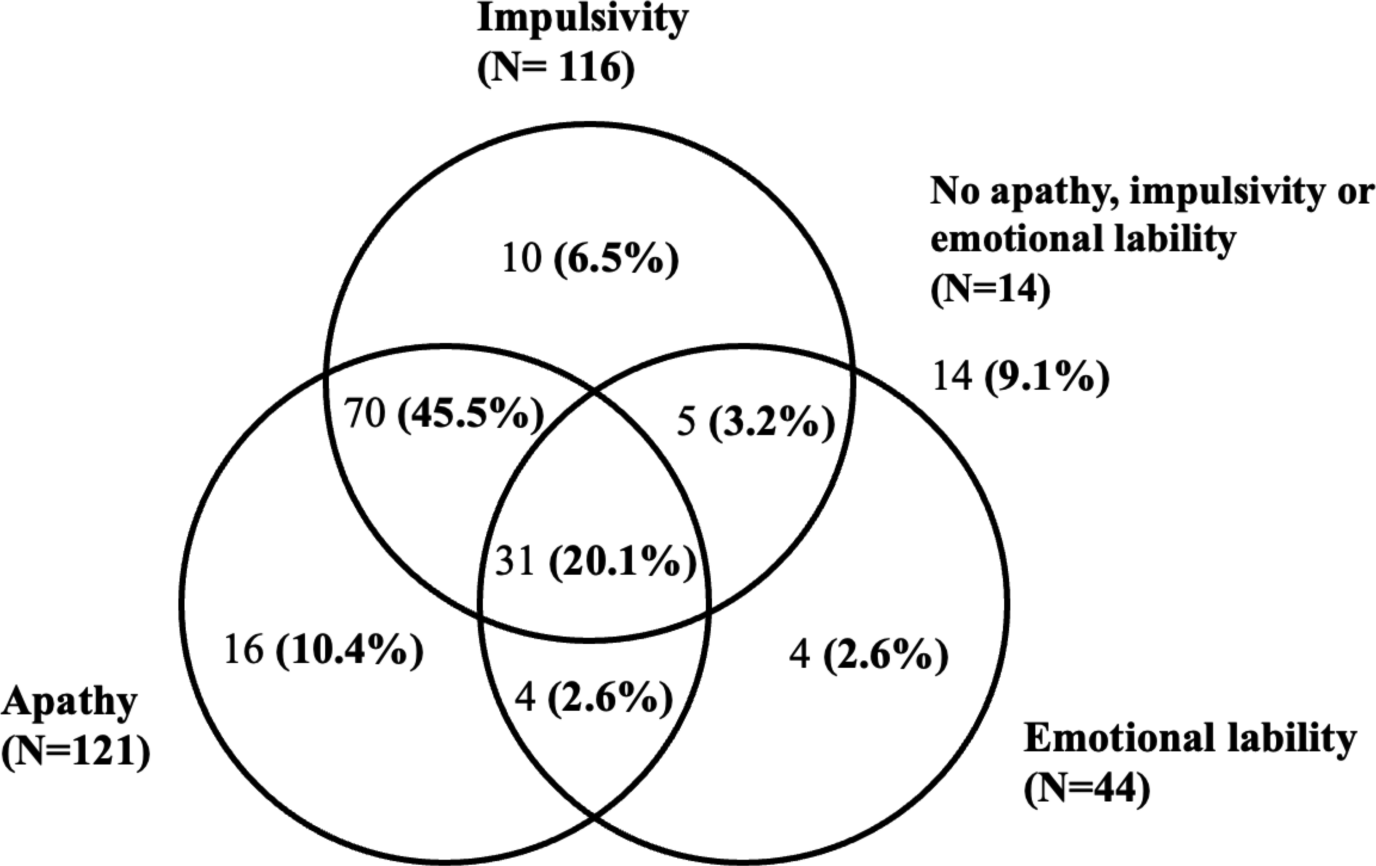
Distribution of patients with apathy, impulsivity and emotional lability (number of patients given, followed by percentage of total patient sample in parentheses).

The prevalence of apathy, impulsivity, and emotional lability is shown in Fig. 1. Overall, 75% of the patient group (n = 116) had impulsive behavioral changes; 79% (n = 121) had apathetic behavioral problems; and 29% of patients (n = 44) manifested emotional lability. Twenty percent displayed all three behavioral changes. Two‐thirds had both apathetic and impulsive behavior reported.

Table 2 shows the relationship between apathy and impulsivity. There was a positive association between them (χ^2^_(1154)_ = 18.2, *P* < 0.001 continuity corrected)). In other words, the probability of being apathetic increases with impulsivity.

**TABLE 2:** Contingency tables for apathy, impulsivity, and emotional lability in PSP patients

Contingency table for apathy and impulsivity. χ^2^_(1154)_ = 18.2, *P* < 0.001 (continuity corrected)				
	Impulsivity (n)			Total (n)
	No		Yes	
Apathy (n)	No	18	15	33
	Yes	20	101	121
Total	38		116	154

Contingency table for emotional lability and impulsivity. χ^2^_(1154)_ < 1, ns (continuity corrected)				
	Emotional lability (n)			Total (n)
	No		Yes	
Impulsivity (n)	No	30	8	38
	Yes	80	36	116
Total (n)	110		44	154

Contingency table for emotional lability and apathy. χ^2^_(1154)_ < 1 (corrected)				
	Emotional lability (n)			Total (n)
	No		Yes	
Apathy (n)	No	24	9	33
	Yes	86	35	121
Total	110		44	154

Contingency table for co‐existent apathy, impulsivity, and emotional lability. χ2_(1154)_ < 1, ns (corrected).				
	Emotional lability (n)			Total (n)
	No		Yes	
Co‐existent apathy and impulsivity (n)	No	41	13	54
	Yes	69	31	100
Total (n)	110		44	154

Table 2 also shows the relationship between emotional lability and impulsivity. There was no significant relationship (*χ* ^*2*^_*(1154)*_ *< 1*, *ns)*. In other words, the probability of being impulsive was independent of emotional lability. Table 2 illustrates the relationship between emotional lability and apathy. There was no significant relationship (*χ* ^*2*^_*(1154)*_ *< 1*, *ns)*. In other words, the probability of being apathetic was independent of emotional lability. Finally, Table 2 shows the relationship between emotional lability and co‐existent apathy and impulsivity. There was no significant relationship (*χ* ^*2*^_*(1154)*_ *< 1*, *ns)*.

**FIG. 2:**
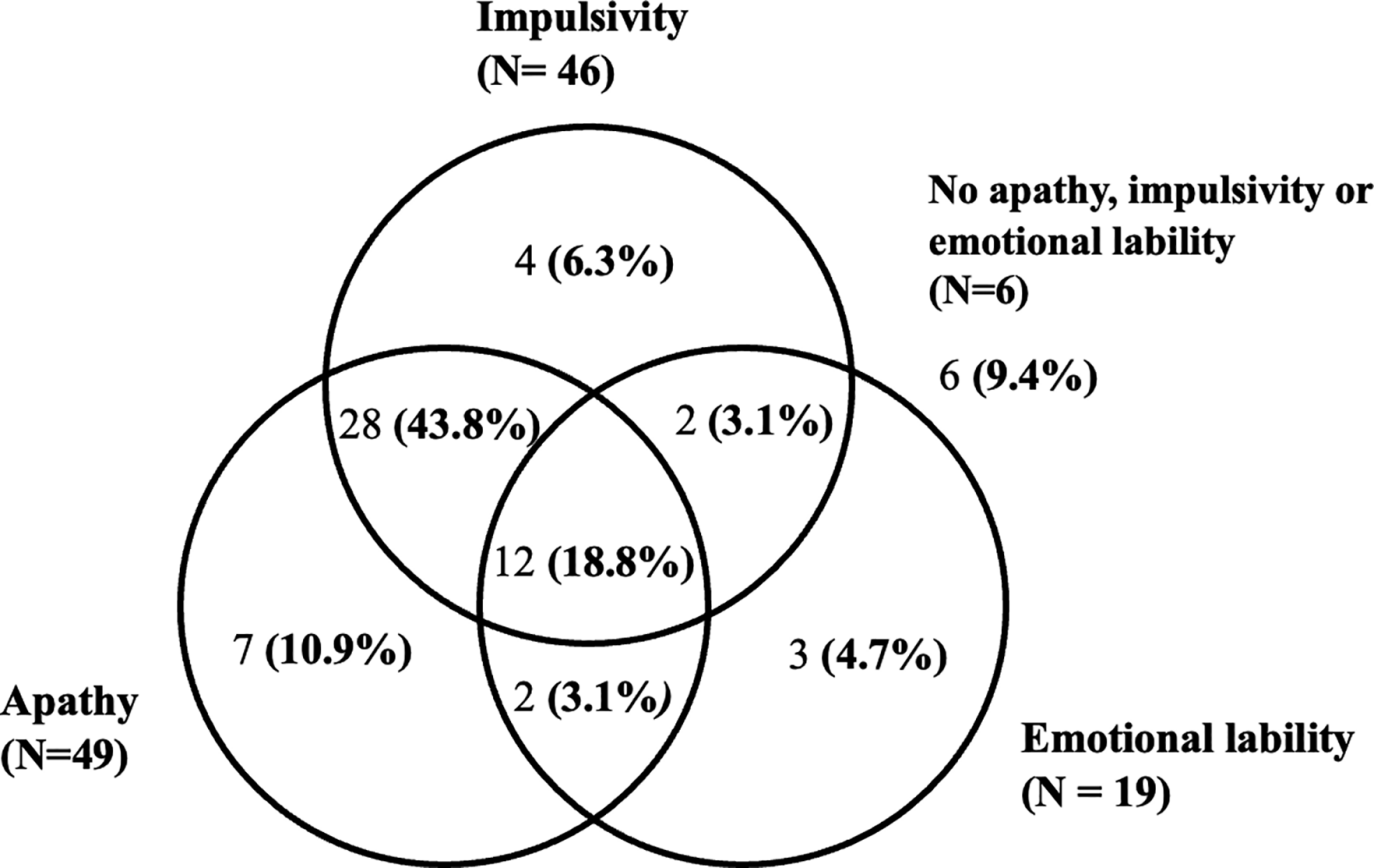
Distribution of behavioral abnormalities in patients with pathological confirmation of PSP (number of patients given, followed by percentage of total patient sample).

There were sixty‐four patients who had pathological confirmation of PSP. The prevalence of apathy, impulsivity, and emotional lability in the patient cohort with pathological confirmation of PSP is shown in Fig. 2. Overall, 72% of those with pathological confirmation of PSP (n = 46) had impulsive behavioral changes; 77% (n = 49) had apathetic behavioral problems; and 30% of patients (n = 19) manifested emotional lability. 19% displayed all three behavioral changes, and 62.6% had both apathetic and impulsive behavior reported. Table 3 illustrates a positive association between apathy and impulsivity in PSP patients with pathological confirmation of diagnosis (*χ* ^*2*^_*(1154)*_ *= 7.89*, *P < 0.005)*. Table 3 also shows that there is no significant relationship between emotional lability and impulsivity in these patients *(χ* ^*2*^_*(1154)*_ *< 1*, *ns)* and that there is no significant relationship between emotional lability and apathy in this patient group *(χ* ^*2*^_*(1154)*_ *< 1*, *ns)*. These results confirm our findings in the main cohort. This is further illustrated in Appendix S7.

**TABLE 3:** Contingency tables for apathy, impulsivity, and emotional lability in PSP patients with pathological confirmation of diagnosis

Contingency table for apathy and impulsivity. χ^2^_(1154)_ = 7.89, *P* < 0.005 (continuity corrected)				
	Impulsivity (n)			
	No		Yes	Total (n)
Apathy (n)	No	9	6	15
	Yes	9	40	49
Total	18		46	64

Contingency table for emotional lability and impulsivity. χ^2^_(1154)_ < 1, ns (continuity corrected)				
	Emotional lability (n)			Total (n)
	No		Yes	
Impulsivity (n)	No	13	5	18
	Yes	32	14	46
Total (n)	45		19	64

Contingency table for emotional lability and apathy. χ^2^_(1154)_ < 1 (corrected)				
	Emotional lability (n)			Total (n)
	No		Yes	
Apathy (n)	No	10	5	15
	Yes	35	14	49
Total	45		19	64

Contingency table for co‐existent apathy, impulsivity, and emotional lability. χ2_(1154)_ < 1, ns (corrected).				
	Emotional lability (n)			Total (n)
	No		Yes	
Co‐existent apathy and impulsivity (n)	No	17	7	24
	Yes	28	12	40
Total (n)	45		19	64

Comparing groups with apathy, impulsivity, neither or both with the frequentist tests (Table 1), we found no significant difference by sex, age at onset, disease duration, disease severity (as indexed by the PSPRS), global cognitive screening tests (ACE‐R, FAB). Bayesian analyses confirmed these null findings with positive or very strong evidence in favor of the null hypothesis (BF_10_ < 1/3 or < 1/10 respectively; Supplementary Materials). We also found no significant between‐groups differences in the PSPRS sub‐scores, except for mentation. Upon further analyses of the data, we found that higher mentation PSPRS sub‐scores related to a higher likelihood of displaying impulsivity, but not apathy (*P* < 0.05).

## Discussion

This study confirms the high frequency of reported co‐existence of apathy and impulsivity in PSP, with a positive relationship between them. Neither apathy nor impulsivity were associated with emotional lability. Approximately three‐quarters of people with PSP had apathy, in keeping with previous reports that apathy is the most common neuropsychiatric feature in PSP.^2^, ^3^, ^4^ Most patients with apathy also manifested impulsive behaviors, more than by chance. The prevalence of impulsivity in our cohort was higher than previously reported (74% versus 32% to 43%^3^, ^4^, ^23^). This disparity may be due to methodological differences to assess impulsivity across studies: patient self‐ratings, clinician judgment of behavioral changes, or carer reports using tools such as the Cambridge Behavioral Inventory (CBI), Neuropsychiatric Inventory (NPI) or Frontal Behavioral Inventory (FBI).

The strong concomitance of apathy and impulsivity accords with previous studies of other syndromes associated with frontotemporal lobar degeneration^9^, ^12^, ^22^, ^27^ and Parkinson's disease.^9^ Such a positive relationship across multiple disorders suggests that apathy and impulsivity may share similar risk factors and etiopathogenetic mechanisms.^22^, ^28^, ^29^ For example, apathy and impulsivity have common correlates in the white‐matter tracts connecting the prefrontal cortex, basal ganglia, temporal poles, and brainstem^22^ as well as gray‐matter atrophy across the frontal cortex.^12^, ^28^ Despite molecular pathological differences, there is convergence onto similar neural circuits across different neurodegenerative disorders. This signals the need to study apathy and impulsivity together rather than in isolation,^11^, ^12^ and highlights the potential for joint therapeutic strategies, rather than dopaminergic antagonism between apathy (or akinesia) and impulsivity.^30^ In view of their positive association, apathy and impulsivity cannot be simply conceptualized as opposite extremes of a dopamine‐dependent spectrum, with apathy arising from a hypodopaminergic state and impulsivity from a hyperdopaminergic state.^14^, ^15^

Other neurotransmitters are likely to influence apathy and impulsivity. For example, serotonin is reduced in several neurodegenerative disorders, and serotonergic manipulations through serotonin reuptake inhibition can ameliorate deficits in response inhibition in patients with Parkinson's disease and frontotemporal dementia.^18^, ^20^ Apathy and impulsivity may also be attributed to noradrenergic deficits.^31^ For example, noradrenergic reuptake inhibition improves response inhibition and restores the function of inhibitory control networks in Parkinson's disease.^19^, ^32^

Emotional lability was not positively associated with either apathy or impulsivity. This suggests that emotional lability arises from dysfunctions in separate neural systems, even though it was relatively common in PSP (with a prevalence of 20%). For example, whilst fronto‐striatal systems are affected in apathy and impulsivity,^11^ fronto‐ponto‐cerebellar circuits have been linked to emotional lability.^33^ Both of these neuroanatomical systems show diffuse tau pathology and neurodegeneration in PSP.

With the exception of the mentation component of the PSPRS subscale, there were no specific demographic or clinical characteristics linked to apathy and impulsivity in PSP. This is in contrast with some studies showing an association between apathy and executive functions.^4^, ^34^ However, another single study did not report such association.^23^ It was also surprising that the Frontal Assessment Battery did not distinguish between patients with and without behavioral changes. A possibility is that the power of our study was insufficient to detect this effect, we therefore do not rule out a potential correlation between executive function and apathy or impulsivity. Some of the mental symptoms described in the PSPRS, such as disorientation, bradyphrenia and utilizing behaviors related to impulsivity in PSP and may be are mediated by overlapping brain networks. Although it is difficult to directly compare studies using different methodologies and assessment tools, the discrepancies may also depend on specific subcomponents of apathy and impulsivity,^11^, ^12^ which we did not differentiate in this study.^3^, ^4^, ^34^ Furthermore, while apathy, impulsivity and executive functions may be mediated by shared fronto‐striatal circuits, progressive dysfunction of the circuits involved may not occur in parallel in PSP.

Our work has limitations. It is a retrospective analysis, albeit drawing on patients with PSP in longitudinal observational studies. We relied on clinical diagnosis, although n = 64 patients had pathological confirmation of PSP diagnosis, and clinicopathological confirmation is typically very high in PSP. Indeed, in this cohort, only two patients received a pathological diagnosis that differed from their clinical diagnosis of PSP. We also included reports of behavioral features over multiple clinical follow‐ups, as it may be that apathy and impulsivity are intermittent rather than constant features. Higher temporal resolution of assessments would be needed to confirm this. Such assessments may rely on carer reports, given a lack of insight into behavioral changes, which can be present in PSP.

We recognize that the qualitative assessment of apathy and impulsivity is an important limitation of our study. Nevertheless, the qualitative coding of apathy and impulsivity was well concordant with the coding of behavioral changes from the PSPRS and CBI scores (see Tables in Appendix S5). In addition, qualitative and quantitative scales have their own strengths and weaknesses. Quantitative scales such as CBI are more generalisable across studies relative to qualitative data such as clinical letters, and provide more details on the severity of behavioral abnormalities. On the other hand, clinical letters contain assessment from multiple informants, including patients, carers, and doctors. For this reason, clinical letters are less vulnerable to variability relating to single informants, or other factors such as carer distress. In our tertiary clinic for PSP, clinical letters emphasize patients' unique perspective, and provide a holistic overview of the various aspects of apathy and impulsivity. They also report real‐life examples of apathy and impulsivity. A potential caveat of using clinical letters is the confirmation bias involved in ascertaining if patients had certain behavioral features, such as apathy and impulsivity. However, we strived to maintain clear selection criteria when assessing these neuropsychiatric problems, drawing on multiple informants and measures. These criteria used the thresholds set out in Appendixes S1–S3. Patients were required to have ≥3 features of apathy, impulsivity or emotional lability, and these features had to be reported persistently across ≥3 clinical follow‐ups.

Another limitation of our study is the dichotomic assessment of apathy and impulsivity, rather than the use of continuous ratings. This was pragmatic and enabled data to be drawn from a larger cohort, and over a longer period of time. Our cross‐sectional findings need to be replicated across other centres and in longitudinal studies and would benefit from systematic measures with a dynamic range suitable for PSP patients. Given the cross‐sectional nature of our findings, it is plausible that we may have underestimated the prevalence of these neuropsychiatric features. However, apathy and impulsivity are often present early in PSP,^28^, ^35^ and our average follow‐up was 5.3 years from symptom onset.

To conclude, our study highlights the co‐morbid nature of apathy and impulsivity in PSP, and their independence from emotional lability. This informs future studies of the neural correlates of apathy and impulsivity, their risk factors, and new therapeutic strategies to reduce apathy and impulsivity.

## Author Roles

(1) Research project: A. Conception, B. Organization, C. Execution; (2) Statistical Analysis: A. Design, B. Execution, C. Review and Critique; (3) Manuscript: A. Writing of the first draft, B. Review and Critique.

ZQK.: 1B, 1C, 2A, 2B, 3A, 3B

AGM.: 2C, 3B

TR.: 2C, 3B

JR.: 1A, 1B, 2C, 3B

LP.: 1A, 1B, 2A, 2C, 3B

## Disclosures

### Ethical Compliance Statement

Written informed consent was obtained from all participants. Ethical approval was given by the East of England ‐ Cambridge Central Research Ethics Committee for the Pick's Disease and Progressive Supranuclear Palsy Prevalence and Incidence protocol (12/EE/0475) (October 2015) and by the East of England—Essex Research Ethics Committee for the Diagnosis and prognosis in Progressive Supranuclear Palsy and Corticobasal Degeneration protocol (07/Q0102/3) (March 2007). All necessary patient/participant consent has been obtained and the appropriate institutional forms have been archived. We confirm that we have read the Journal's position on issues involved in ethical publication and affirm that this work is consistent with those guidelines.

### Funding Sources and Conflicts of Interest

This study was co‐funded by the Cambridge University Centre for Parkinson‐Plus (RG95450) and the Medical Research Council (RG91365). The Cambridge Brain Bank is supported by the NIHR Cambridge Biomedical Research Centre. The authors also declare that there are no conflicts of interest relevant to this work.

### Financial Disclosures for the previous 12 months

James B. Rowe serves as an associate editor to Brain and is a non‐remunerated trustee of the Guarantors of Brain, Darwin College and the PSP Association (UK). He provides consultancy to Asceneuron, Biogen, UCB and has research grants from AZ‐Medimmune, Janssen, Lilly as industry partners in the Dementias Platform UK. The authors declare that there are no additional disclosures to report.

## Supporting information


**Appendix S1.** Qualitative selection criteria for apathy, impulsivity and emotional lability (linguistic analysis of clinical letters).
**Appendix S2.** Cambridge Behavioral Inventory score thresholds for apathy and impulsivity.
**Appendix S3.** PSP‐RS (Progressive Supranuclear Palsy‐Rating Scale) score thresholds for apathy and impulsivity.
**Appendix S4.** Distribution of CBI subscores representing apathy and impulsivity.
**Appendix S5.** Concordance between encoding of behavioral changes from clinical letters, CBI and PSP‐RS clinical scores.
**Appendix S6.** Average CBI subscores of patients with and without apathy and impulsivity.
**Appendix S7.** Pathological confirmation of PSP in behavioral subgroups.
**Appendix S8.** Bayesian ANOVAs were used to examine differences between clinical variables across the behavioral subgroups.
