# Supplementary material for: Co‐Occurrence of Apathy and Impulsivity in Progressive Supranuclear Palsy
Source: Mov Disord Clin Pract. 2021 Sep 16;8(8):1225–33. doi: 10.1002/mdc3.13339 (PMC8564809; doi:10.1002/mdc3.13339)
Supplement: Supplementary file 1 — Appendix S1. Qualitative selection criteria for apathy, impulsivity and emotional lability (linguistic analysis of clinical letters). Appendix S2. Cambridge Behavioral Inventory score thresholds for apathy and impulsivity. Appendix S3. PSP‐RS (Progressive Supranuclear Palsy‐Rating Scale) score thresholds for apathy and impulsivity. Appendix S4. Distribution of CBI subscores representing apathy and impulsivity. Appendix S5. Concordance between encoding of behavioral changes from clinical letters, CBI and PSP‐RS clinical scores. Appendix S6. Average CBI subscores of patients with and without apathy and impulsivity. Appendix S7. Pathological confirmation of PSP in behavioral subgroups. Appendix S8. Bayesian ANOVAs were used to examine differences between clinical variables across the behavioral subgroups. [file MDC3-8-1225-s001.docx]

**Supplementary Materials**

**Bayesian ANOVAs**

We compared subgroups with ‘both apathy and impulsivity’, ‘only apathy, no impulsivity’, ‘only impulsivity, no apathy’, and ‘neither apathy nor impulsivity’. Bayesian ANOVAs were used to examine differences between clinical variables across the various behavioural subgroups. In this study, the null hypothesis (H_0_) is that there is no difference between the behavioural subgroups for the relevant clinical or disease variable. The alternative hypothesis (H_1_) is that there is a difference between the behavioural subgroups for the relevant disease or clinical variable. Priors assumed equipoise between H_0_ and H_1_. The hypotheses are compared in terms of the Bayes factor, BF. The BF_10_ for the null models was >0.99, indicating that the null model was consistently the best model. The Bayes factors indicated very strong evidence in favour of the null model for differences in ACE-R and FAB (BF_01_>10), but there was inconclusive evidence between models for PSPRS (1/3>BF>3).

**Appendix 1: Qualitative selection criteria for apathy, impulsivity and emotional lability (linguistic analysis of clinical letters)**

In order for these behavioural changes to be encoded, patients were required to have 3 or more recorded qualitative features of apathy, impulsivity or emotional lability, and these features had to be reported persistently across 3 or more clinical follow-ups.

| **Qualitative descriptions of apathy that met the selection criteria** | **Number of patients with description** |
| --- | --- |
| Apathetic/Showed apathy (most common) | 102 |
| Reduced or loss of motivation/loss of initiative | 98 |
| Reduced/Lost interest in new activities | 57 |
| Social withdrawal i.e. more socially withdrawn/no longer engages in conversations around him/her | 78 |
| Loss of enjoyment and interest in seeing friends/doing things he/she used to enjoy” | 50 |
| Less conversational | 10 |
| Shows reduced affection | 21 |
| More mellow personality | 10 |
| **Total number of patients** | 121 |

| **Qualitative descriptions of impulsivity that met the selection criteria** | **Number of patients with description** |
| --- | --- |
| “Impulsivity”“impulsive behaviour” (most common) | 102 |
| Marked/significant disinhibition | 80 |
| **Social behaviours** | |
| Socially or sexually inappropriate behaviours/comments | 53 |
| Coarser humour | 20 |
| Aggressive behaviour towards family members/other people (physically and/or verbally) | 25 |
| More bloody minded, short-tempered and/or more argumentative and/or more temper outbursts | 12 |
| **Eating behaviours** | |
| Compulsive eating behaviours/Cramming behaviours (sometimes leading to dysphagia and choking) +/- decline in table manners | 75 |
| Hyperphagia/hyperorality  Examples:  “Tried to swallow large pieces of food instead of cutting and chewing”  “Tendency to overfill his mouth, stuffing his mouth and sucking ++ on beaker” | 30 |
| Developed/Increasing sweet tooth | 22 |
| **Motor behaviours** | |
| Unsafe behaviour | 23 |
| Motor impulsivity/recklessness and delay intolerannce | 38 |
| Impulsive decision-making  Examples:  “Making impulsive and irrational decisions that put him at risk”  “ Rushes into things or jumps to conclusions and makes decisions without regard for consequences” | 13 |
| **Total number of patients** | 116 |

| **Qualitative descriptions of emotional lability that met the selection criteria** | **Number of patients with description** |
| --- | --- |
| Emotional lability | 35 |
| Emotional incontinence | 22 |
| Pseudobulbar affect | 28 |
| Physical manifestations of distress and crying without corresponding inner emotions | 10 |
| Emotional volatility  Examples:  “Can get angry or burst into tears without warning”  “Can get tearful or emotional on frequent occasions without any clear triggers to this” | 16 |
| Emotional incongruence  Examples:  “Unexplained bursting into tears and appearing distressed despite not being internally distressed”  “Tears without feeling sad”  “Laughing and crying without feeling happy or sad” | 15 |
| Laughing/crying out of context/ Inappropriate laughing or crying | 20 |
| **Total number of patients** | 44 |

**Appendix 2: Cambridge Behavioural Inventory score thresholds for apathy and impulsivity**

**CBI Score Thresholds for Apathy**

- Total score of 1 or more on the *Motivation* section of the Cambridge Behavioural Inventory is defined as the presence of apathy, as below:

**
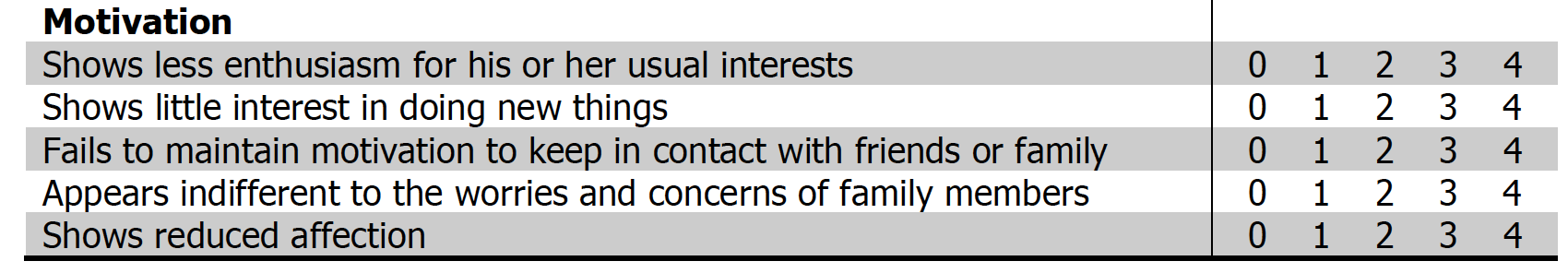
**^37^

**CBI Score Thresholds for Impulsivity**

- Total score of 1 or more on the *Abnormal Behaviour* section of the Cambridge Behavioural Inventory **and/or**
- Total score of 1 or more on the subsections *“Her/his appetite is greater, s/he eats more than before”* and/or *“Table manners are declining”*, within the *Eating Habits* section of the Cambridge Behavioural Inventory is defined as the presence of impulsivity

As below:

**
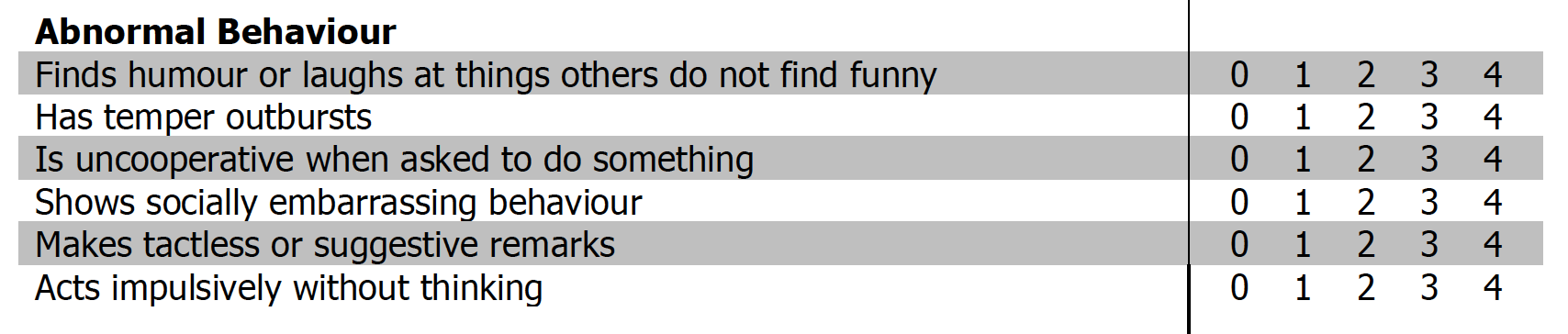
** **
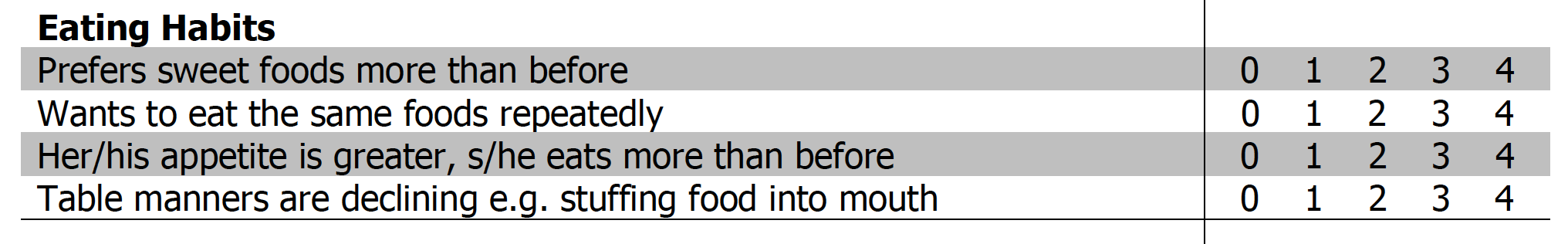
**


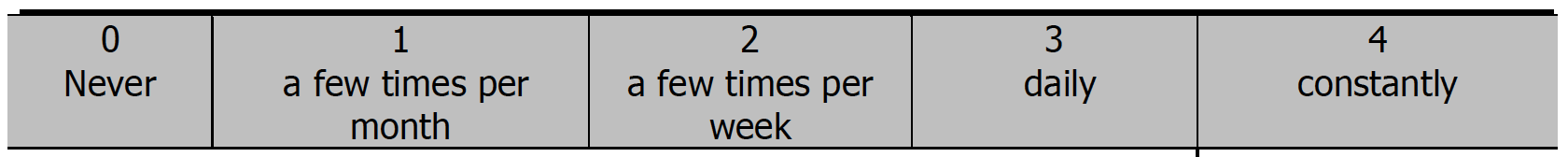


^37^

**Appendix 3: PSP-RS (Progressive Supranuclear Palsy- Rating Scale) score thresholds for apathy and impulsivity**

**Progressive Supranuclear Palsy - Rating Scale Score Thresholds for Apathy**

- Total score of 1 or more on the *Withdrawal* section of the PSP-RS is defined as the presence of apathy, as below:

**
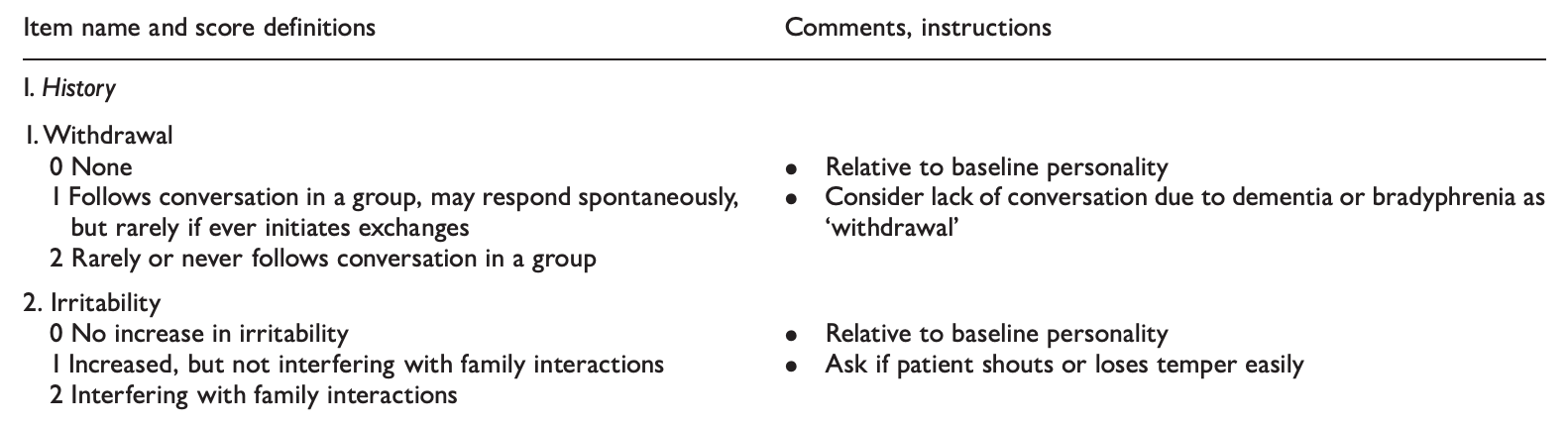
**^38^

**Progressive Supranuclear Palsy - Rating Scale Score Thresholds for Impulsivity**

- Total score of 1 or more on the *Irritability* section of PSP-RS is defined as the presence of impulsivity, as below:

**
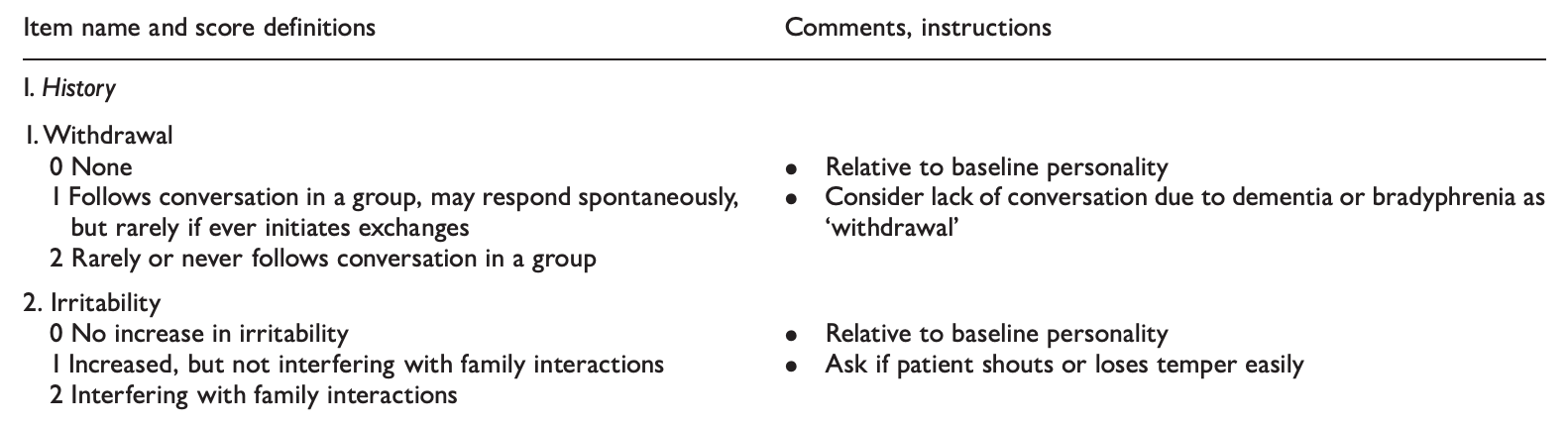
**

^38^

**Appendix 4**


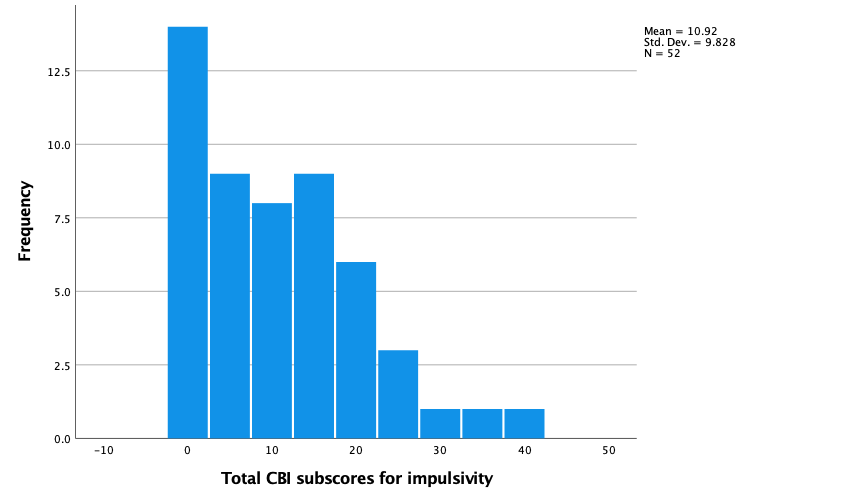


**Distribution of CBI subscores**


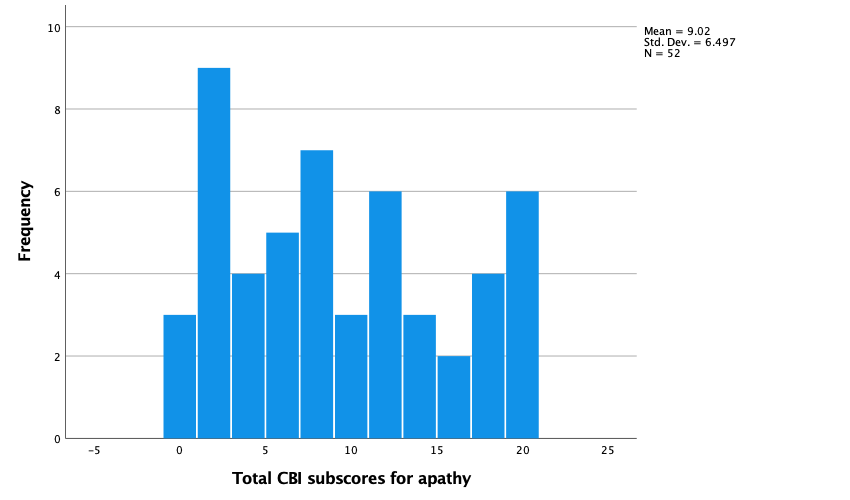


**Distribution of CBI subscores**

**Appendix 5: Concordance between encoding of behavioural changes from clinical letters, CBI and PSP-RS clinical scores**

**Total number of patients with PSP-RS scores available: 79**

|  | Patients coded as apathetic from PSP-RS subscores (n=63) | Patients coded as non-apathetic from PSP-RS subscores (n=16) |
| --- | --- | --- |
| Patients coded as apathetic from clinical letters | 63 | 0 |
| Patients coded as non- apathetic from clinical letters | 0 | 16 |
| % concordance rate between coding from PSP-RS and clinical letters | 100 | 100 |

|  | Patients coded as impulsive from PSP-RS subscores (n=59) | Patients coded as non-impulsive from PSP-RS subscores (n=20) |
| --- | --- | --- |
| Patients coded as impulsive from clinical letters | 59 | 0 |
| Patients coded as non- impulsive from clinical letters | 0 | 16 |
| % concordance rate between coding from PSP-RS and clinical letters | 100 | 100 |

**Total number of patients with CBI scores available: 52**

|  | Patients coded as apathetic from CBI subscores (n=49) | Patients coded as non-apathetic from CBI subscores (n=3) |
| --- | --- | --- |
| Patients coded as apathetic from clinical letters | 49 | 0 |
| Patients coded as non- apathetic from clinical letters | 0 | 3 |
| % concordance rate between coding from PSP-RS and clinical letters | 100 | 100 |

|  | Patients coded as impulsive from CBI subscores (n=46) | Patients coded as non-impulsive from CBI subscores (n=6) |
| --- | --- | --- |
| Patients coded as impulsive from clinical letters | 46 | 0 |
| Patients coded as non-impulsive from clinical letters | 0 | 6 |
| % concordance rate between coding from PSP-RS and clinical letters | 100 | 100 |

|  | Patients coded as apathetic from CBI subscores (n=49) | Patients coded as non-apathetic from CBI subscores (n=3) |
| --- | --- | --- |
| Patients coded as apathetic from clinical letters | 49 | 0 |
| Patients coded as non- apathetic from clinical letters | 0 | 3 |
| % concordance rate between coding from PSP-RS and clinical letters | 100 | 100 |

**Appendix 6**

**CBI subscores of patients with and without impulsivity (p<0.001)**

|  | Number of patients | Mean | Standard Deviation |
| --- | --- | --- | --- |
| Patients who were coded as impulsive from CBI | 46 | 12.35 | 9.564 |
| Patients who were coded as non-impulsive from CBI | 6 | .00 | .000 |

**CBI subscores of patients with and without apathy (p=0.004)**

|  | Number of patients | Mean | Standard Deviation |
| --- | --- | --- | --- |
| Patients who were coded as apathetic from CBI | 49 | 9.57 | 6.282 |
| Patients who were coded as non-apathetic from CBI | 3 | .00 | .000 |

**Appendix 7: Pathological confirmation of PSP in behavioural subgroups**

| **Behavioural Subgroup** | **Number of patients with pathological confirmation** | **% of cohort with pathological confirmation of PSP** | **% of total cohort** |
| --- | --- | --- | --- |
| Both apathy and impulsivity, no emotional lability | 28 | 43.8 | 45.5 |
| Impulsivity alone | 4 | 6.3 | 6.5 |
| Apathy alone | 7 | 10.9 | 10.4 |
| None of apathy, impulsivity or emotional lability | 6 | 9.4 | 9.1 |
| Patients who have apathy, impulsivity and emotional lability | 12 | 18.8 | 20.1 |
| Emotional lability alone | 3 | 4.7 | 2.6 |
| Both apathy and emotional lability, no impulsivity | 2 | 3.1 | 2.6 |
| Both impulsivity and emotional lability, no apathy | 2 | 3.1 | 3.2 |
| Total | 64 | 100 | 100 |
